# DOES SLC11A2 GENE MUTATION ASSOCIATE WITH IRON-REFRACTORY IRON-DEFICIENCY ANEMIA AFTER BARIATRIC SURGERY?

**DOI:** 10.1590/0102-672020210002e1645

**Published:** 2022-06-17

**Authors:** Vânia Belintani PIATTO, Danielle Lopes Teixeira FERDINANDO, Hamilton Luiz Xavier FUNES

**Affiliations:** 1 Faculty of Medicine, São Jose do Rio Preto, Department of Anatomy - São José do Rio Preto - São Paulo - Brazil;; 2 Faculty of Medicine, São José do Rio Preto, Department of Pediatrics and Pediatrics Surgery - São José do Rio Preto - São Paulo - Brazil.

**Keywords:** Anemia, Iron-Deficiency, Anemia, Refractory, Bariatric Surgery, Molecular Biology, Mutation, Anemia Ferropriva, Anemia Refratária, Cirurgia Bariátrica, Biologia Molecular, Mutação

## Abstract

After bariatric surgery, if there is iron-refractory iron-deficiency anemia (IRIDA) and does not respond to supplemental iron therapy, excluding other possible etiologies, genetic changes involved in iron metabolism should be considered.

**AIM:**

This study aimed to investigate the association of both mutations 1285G-C and 1246C-T, in the *SLC11A2* gene, and the etiopathogenesis of anemia refractory to iron supplementation in patients undergoing bariatric surgery using Roux-en-Y gastric bypass (RYGB).

**METHODS:**

A case-control study was conducted, in which 100 patients were evaluated as Cases Group [subdivided into (i) with Anemia and (ii) without Anemia] and 100 individuals as Controls, comprising both sexes. Inherited and acquired causes of IRIDA were excluded. DNA was extracted from leukocytes of peripheral blood, and the regions that cover both mutations have been amplified by the molecular techniques such as polymerase chain reaction/restriction fragment length polymorphism.

**RESULTS:**

The 1285G-C mutation was not determined in any of the 400 alleles analyzed. Regarding the 1246C-T mutation, the wild CC genotype was found with a higher prevalence in the Control Group (34%) (OR 0.5475; 95%CI 0.2920-1.027; p=0.0827). The mutant TT genotype was found only in the Cases Group I (with Anemia) (13%).

**CONCLUSION:**

The results show the association between 1246C-T mutation, in the *SLC11A2* gene, and the etiopathogenesis of IRIDA to iron supplementation in the evaluated sample. There are differences, at the molecular level, in patients with and without IRIDA after bariatric surgery using RYGB.

## RESUMO - RACIONAL

Após cirurgia bariátrica, se houver anemia por deficiência de ferro e não responder à terapia de ferro suplementar, excluindo-se outras possíveis etiologias, alterações genéticas envolvidas no metabolismo férrico devem ser consideradas.

**OBJETIVO:**

Investigar a associação das mutações 1285G-C e 1246C-T, no gene *SLC11A2*, e a etiopatogênese da anemia refratária à suplementação de ferro em pacientes submetidos à cirurgia bariátrica pela técnica de derivação gástrica em Y-de-Roux.

**MÉTODOS:**

Estudo de caso-controle, no qual forma avaliados 100 pacientes em Grupos de Casos (subdividido em Grupo I - com Anemia e Grupo II - sem Anemia) e 100 indivíduos como Controles, de ambos os sexos. Causas hereditárias e adquiridas de anemia ferropriva refratária ao ferro, foram excluídas. O *DNA* foi extraído de leucócitos de sangue periférico e as regiões que abrangem ambas as mutações foram amplificadas pelas técnicas moleculares de Reação em Cadeia da Polimerase/Polimorfismo do Comprimento do Fragmento de Restrição.

**RESULTADOS:**

A mutação 1285G-C não foi determinada em quaisquer dos 400 alelos analisados. Em relação à mutação 1246C-T, o genótipo homozigoto selvagem CC foi encontrado com maior prevalência nos Controles (34%) (OR: 0,5475; 95%IC: 0,2920-1,027; p=0,0827). O genótipo homozigoto mutante TT foi encontrado apenas no Grupo I - com Anemia (13%).

**CONCLUSÃO:**

Os resultados demonstram a associação da mutação 1246C-T, no gene *SLC11A2,* e a etiopatogênese da anemia ferropriva refratária e persistente à suplementação de ferro, nesta amostra de pacientes. Há diferenças, em nível molecular, em pacientes com e sem anemia ferropriva refratária ao ferro após cirurgia bariátrica por derivação gástrica em Y-de-Roux.

## INTRODUCTION

The surgical treatment of obesity has become the main alternative for weight control using Roux-en-Y gastric bypass (RYGB) surgery as one of the most widespread techniques^15^. However, the clinical follow-up after bariatric surgery has shown a tendency toward hematological alterations, especially chronic anemia, in addition to nutritional alterations^5^ ^,^ ^16^ ^,^ ^20^. After surgery, anemia due to inappropriately low levels of erythropoietin or iron deficiency is common. Furthermore, suppression of erythropoiesis and interference with the production of erythropoietin or its signaling pathway may contribute to the reduction in red blood cell production. In the long term, the estimated incidence of anemia after RYGB surgery ranges from 12% to 53%, which is mainly attributed to micronutrient deficiency, including the main hematinic factors, iron, and vitamin B12^16^ ^,^ ^20^. As there is still a significant number of iron-refractory iron-deficiency anemias (IRIDAs) unresponsive to iron supplementation therapy, which remain unexplained after RYGB surgery, the hypothesis of genetic alterations involved in iron metabolism should be considered. In addition to the recommended hematological and biochemical parameters^2^ ^,^ ^4^ ^,^ ^6^ ^,^ ^10^ ^,^ ^13^ ^,^ ^19^ ^,^ ^20^, molecular investigation should be carried out.

Genetic alterations related to IRIDA involve mutations in genes that play essential roles in systemic or cellular iron metabolism. Among the several genes involved, the *SLC11A2* gene (Solute Carrier Family 11, member A2), located on chromosome 12 (12q13.12), is responsible for encoding the divalent metal transporter 1 (DMT-1) protein, which is expressed in the apical membrane of enterocytes and in the endosomes of duodenal cells and peripheral tissues. The DMT-1 protein transports not only iron, as it is better known, but also copper, manganese, cadmium, and zinc, from the duodenal luminal surface to the cell interior. Dietary heme iron is transported across the enterocyte by heme carrier protein 1 (HCP-1)^13^. Genetic alterations in the DTM-1 protein may cause damage not only to the luminal absorption of iron into the enterocyte but also to the absorption of copper, which will not be incorporated into copper-dependent enzymes, such as hephaestin and ceruloplasmin, that are responsible for the oxidation of the iron so that it binds to transferrin and is transported into tissues. Both enzymes, with inadequate incorporation of copper, will not convert iron to the ferric state and cannot link to transferrin for transport into tissues. Consequently, the clinical profile of anemia is related to the dual role of the DMT-1 protein in iron and copper absorption^3^. However, copper is also absorbed in the apex of enterocytes by the copper transporter receptor-1 (CTR-1) protein, thus not causing changes in its absorption and functions^11^ ^,^ ^13^.

Among the mutations identified in the *SLC11A2* gene, which encodes the DMT-1 protein, two were more associated with the clinical condition of microcytic hypochromic anemia of a genetic cause, due to changes in protein conformation: (1) The 1285G-C transversion (IVS12ds-1 GC), in exon 12/intron 12, which results in the replacement of glutamic acid by aspartate, by changing the GAG codon to GAC, at position 399 (Glu399Asp; E399D)^9^; (2) the 1246C-T transition, in exon 13, by changing the CGC codon to TGC, at position 416, resulting in the replacement of arginine by cysteine (Arg416Cys; R416C)^8^. Therefore, mutations in this gene cause decreased iron utilization by erythroblasts, although hepatic iron stores are increased. This clinical profile is related to the dual role of DMT-1 protein in dietary iron uptake at the apical membrane of the duodenal enterocyte and in iron egress from cytosolic endosomes. Apparently, there is a relationship between phenotype and genotype. Thus, a patient with a mutation that modifies the function, without altering the protein expression, has milder anemia than the one with a mutation that alters the amount of protein^3^ ^,^ ^8^ ^,^ ^9^ ^,^ ^14^.

The study aimed to investigate the association of both mutations 1285G-C and 1246C-T, in the *SLC11A2* gene, and the etiopathogenesis of IRIDA to iron supplementation in patients undergoing bariatric surgery using RYGB.

## METHODS

A retrospective case-control study was conducted by using demographic and laboratory data from (1) Cases Group - 100 patients comprising both sexes, aged 18-65 years, who underwent bariatric surgery using RYGB, from 2016 to 2019, at the Institution, and who have been in clinical-nutritional follow-up for more than 1 year and (2) Control Group - 100 patients comprising both sexes, aged 18-65 years, not undergoing bariatric surgery.

The Cases Group was subdivided into Group I (with Anemia) and Group II (without Anemia). For the selection and inclusion of patients in the Groups, the following criteria were considered: have undergone bariatric surgery using RYGB; presence (Group I) or not (Group II) of IRIDA to post-surgery iron supplementation; absence of inherited anemia; laboratory tests (hematological, biochemical), and radiological and/or endoscopic exams, which are part of the pre- and post-surgical protocol established by the Brazilian Society of Bariatric and Metabolic Surgery (2018), regardless of parallel scientific research, without any alterations. As exclusion criteria, the following were considered: bariatric surgery performed by a standardized technique other than RYGB; other causes of blood loss (female patients with metrorrhagia; gastrointestinal or uropelvic malignancy; bleeding from anastomotic ulcers; the presence of small intestinal bacterial overgrowth, *Helicobacter pylori*, iron losing-enteropathy, chronic inflammation, myelodysplastic syndromes, hematological malignancies, etc.); patients with complications after surgery (fistula, malnutrition, mineral and vitamin deficiencies, leak of anastomosis).

A total of 100 patients in the Control Group were included using the following criteria: not undergoing bariatric surgery, clinical and laboratory tests within normal standards, and absence of comorbidities and/or history of malignancies.

The following demographic data were collected for both groups: sex, age at the time of surgery (Cases Group), and age at the time of inclusion in the study (Control Group). Anemia was defined as a serum hemoglobin value <13.5 g/dL in males and <12.0 g/dL in females, per usual laboratory parameters. Patients and controls are of the same ethnicity/skin color (i.e., Caucasians) and of the same geographical area (i.e., State of São Paulo, Brazil).

### Molecular Analysis

Genomic DNA was isolated from peripheral blood leukocytes using the GE Illustra - Blood GenomicPrep Mini Spin Kit^™^ (GE Healthcare UK Limited^™^) using the manufacturer’s protocol, and the procedure was performed in the Teaching and Research Sector of the Institution's Macroscopy Laboratory. The extracted DNA was stored at 4°C for 24 h before being frozen at −20°C.

To detect both 1285G-C and 1246C-T mutations (OMIM - NM_000617.2), genomic DNA fragments that encompassed both altered sites, in the *SLC11A2* gene (OMIM ID*600523), were amplified by polymerase chain reaction (PCR), using PCR Reagent Kit - OneTaq^®^Hot Start Quick-Load^®^ 2X Master Mix with Standard Buffer (New England Biolab^™^), in a final volume of 25 μL, with previously published primers to the respective mutations^8^ ^,^ ^9^ and then digested using restriction fragment length polymorphism (RFLP), with 10U, for 1.0 h at 37°C, of their respective restriction enzymes (New England Biolab™) - BsiWI and MlyI.

After the RFLP reaction, the 1285G wild-type allele from *SLC11A2* gene was represented by an uncut 422-base pair (bp) PCR product, and the 1285C mutant allele consisted of two fragments at 374 bp and 48 bp. The 1246C wild-type allele from *SLC11A2* gene consisted of two fragments at 290 bp and 135 bp, and the 1246T mutant allele was represented by an uncut 425-bp PCR product.

The products from both PCR/RFLP reactions were added to FlashGel^™^ Loading Dye 5x, analyzed by electrophoresis on a 1.2% or 2.2% agarose FlashGel^™^ DNA System Cassette to confirm their success, and all gels were photodocumented using FlashGel^™^ Camera (Lonza Group Ltd Muenchensteinerstrasse 38 CH-4002 Basel Switzerland).

To avoid bias in the molecular analysis and final results, all DNA samples were analyzed without knowledge of the patients’ clinical characteristics.

### Ethical Aspects

The ethics and research protocol were approved by the Research Ethics Committee (Faculty of Medicine of São José do Rio Preto - FAMERP, São Paulo, Brazil) (no 2.982.247/2018). Before initiation of any procedure, signed informed consent was obtained from all patients.

### Statistical Analysis

Results were previously submitted to descriptive statistics to determine the normal range. The Mann-Whitney U test was used for independent samples with non-normal distribution, and the unpaired t-test was applied for independent samples with a normal distribution. Fisher’s exact test and/or chi-square were used to compare the variables, wherever applicable. Odds ratio (OR) test with 95% confidence interval (CI) was used. Results were considered statistically significant when the probability of findings occurring by chance was 5% (p<0.05). Qualitative variables were expressed as a percentage (%) and continuous variables were expressed as median (*M*) and standard deviation (SD). Statistical tests were performed using the GraphPad InStat version 3.00 program (GraphPad Software Inc, San Diego, CA, USA).

## RESULTS

Of the 100 patients in the Cases Group, 93 (93%) were females and 7 (7%) were males. Regarding age, it ranged, at the time of bariatric surgery, from 20 to 63 years (*M*=43 years; SD=10.5) for females and from 20 to 63 years (*M*=40 years; SD=15.1) for males, without statistically significant differences (p=0.9495).

In relation to the Control Group, of the 100 patients, 90 (90%) were females and 10 (10%) were males. Regarding age, it ranged from 18 to 65 years (*M*=41 years; SD=13.7) for females and from 25 to 62 years (*M*=42.5 years; SD=13.9) for males, without statistically significant differences (p=0.5200).

As reported, the Cases Group was subdivided into Group I (with Anemia) with 48 patients (48%), i.e., 44 (44%) females and 4 (4%) males, and Group II (without Anemia) composed of 52 patients (52%), i.e., 49 (49%) females and 3 (3%) males, without statistically significant difference (p=0.7075).

### Molecular Results

The 1285G-C mutation, in exon 12/intron 12, in the *SLC11A2* gene, was not found in any of the 200 alleles analyzed in the patients of the Cases Group, as well as in the 200 alleles of the patients in the Control Group. Therefore, the GG genotype (wild homozygote) was determined for the entire sample of both Groups - Cases and Controls (400 alleles - 100%).

Table 1 shows the genotypic and allelic results, which was found in both the Cases and Control Groups, related to the 1246C-T mutation, in exon 13, in the *SLC11A2* gene.

**Table 1 -:** Genotypic and allelic relationship for the 1246C-T mutation, in the *SLC11A2* gene, between Cases and Control Groups.

Gene/mutation	Genotypes/alleles	Cases N=100 (%) Alleles N=200 (%)	Controls N=100 (%) Alleles N=200 (%)	OR (95%CI)	p-value*
*SLC11A2*/1246C-T	CC	22 (22)	34 (34)	0.5475 (0.2920-1.027)	0.0827
	CT	65 (65)	66 (66)	0.9567 (0.5339-1.714)	1.0000
	TT	13 (13)	0 (0)	-	NA
	C	109 (54.5)	134 (67)	0.5900 (0.3933-0.8805)	0.0139
	T	91 (45.5)	66 (33)	1.695 (1.130-2.543)	0.0139

*Fisher’s exact test.

Polymorphic allele and significant results (p<0.05) are mentioned in bold.

N: number; OR: odds ratio; CI: confidence interval; NA: not analyzed.

The wild homozygous CC genotype was found with the highest prevalence in the Control Group (34%) (OR 0.5475; 95%CI 0.2920-1.027; p=0.0827). The mutant homozygous TT genotype was found only in the Cases Group (13%). The wild C allele was significantly associated with approximately 1.69-fold (1/0.59) more to the protection factor to the chance of anemia (OR 0.5900; 95%CI 0.3933-0.8805; p=0.0139). Regarding the mutant T allele, it was significantly associated with the chance of anemia, about 1.69-fold more (OR 1,695; 95%CI 1.130-2.543; p=0.0139).

Table 2 shows the genotypic and allelic results, found between Group I (with Anemia) and Group II (without Anemia), related to the 1246C-T mutation, in exon 13, in the *SLC11A2* gene.

**Table 2 -:** Genotypic and allelic relationship for the 1246C-T mutation, in the *SLC11A2* gene, between the Group I (with Anemia) and Group II (without Anemia).

Gene/mutation	Genotypes/alleles	Group I N=48 (%) Alleles N=96 (%)	Group II N=52 (%) Alleles N=104 (%)	OR (95% CI)	p-value*
*SLC11A2*/1246C-T	CC	7 (15)	15 (29)	0.4211 (0.1547-1.146)	0.0968
	CT	28 (58)	37 (71)	0.5676 (0.2474-1.302)	0.2113
	TT	13 (27)	0 (0)	-	NA
	C	42 (44)	67 (64)	0.4295 (0.2431-0.7589)	0.0044
	T	54 (56)	37 (36)	2.328 (1.318-4.113)	0.0044

*Fisher’s exact test.

Polymorphic allele and significant results (p<0.05) are mentioned in bold.

N: number; OR: odds ratio; CI: confidence interval; NA: not analyzed.

The wild homozygous CC genotype was found to be more prevalent in Group II (without Anemia) (29%) (OR 0.4211; 95%CI 0.1547-1.146; p=0.0968). The mutant homozygous TT genotype was found only in Group I (with Anemia) (13%). The wild C allele was significantly associated with about 2.3-fold (1/0.4295) more to the protection factor to the chance of anemia (OR 0.4295; 95%CI 0.2431-0.7589; p=0.0044). Regarding the mutant T allele, it was significantly associated with the chance of anemia, about 2.3-fold more (OR 2.328; 95%CI 1.318-4.113; p=0.0044).

## DISCUSSION

The genetic causes of IRIDA, real or functional, have allowed us to clarify that the implication of the components of the iron metabolic pathway is due to alterations in some of the diverse proteins involved in the absorption and metabolism of this micronutrient^2^ ^,^ ^3^ ^,^ ^14^ ^,^ ^18^.

Most patients with iron-deficiency anemia, post-bariatric surgery, are efficiently treated with iron supplementations. However, some of these patients are refractory to such treatments, even when the pathological condition underlying the anemia is simultaneously treated. The pathological basis for this refractoriness can be explained by several factors, including malabsorption of iron (atrophic gastritis), deficiency of other hematopoietic vitamins or minerals (vitamin B12, zinc, copper), other undiagnosed anemic disorders (renal anemia or hematopoietic diseases), as well as certain hereditary disorders of iron metabolism, for example IRIDA caused by genetic mutation of the *SLC11A2* gene^14^ ^,^ ^17^ ^,^ ^18^. Among the mutations identified in the *SLC11A2* gene, which encodes the DMT-1 protein, two were more associated with a clinical picture of microcytic hypochromic anemia of genetic cause, due to changes in protein conformation: the 1285G-C transversion, in exon 12/intron 12^9^ and the 1246C-T transition, in exon 13^8^.

To date, there are no data in the literature on the molecular analysis of the *SLC11A2* gene in order to verify the prevalence of the mutations described above in patients with IRIDA, no responsive to therapeutic supplementation, who underwent bariatric surgery using RYGB, and what reinforces the importance of this study.

For the evaluation of results of genetic association, it is important that the research subjects have the same ethnicity, or skin color, and the same geographical origin, since the genetic bases of different diseases may differ between various regions and between populations^21^, which was considered in the present study. Despite the intense Brazilian miscegenation, patients and controls with apparent similarity in skin color and from the same geographical area were included.

In the present study, both 1285G-C and 1246C-T mutations in the *SLC11A2* gene were investigated in patients undergoing bariatric surgery using RYGB, with and without anemia, and in Controls patients. Regarding the 1285G-C mutation, it was not found in all patients of both Cases and Controls Groups, and none of the patients were born from consanguineous marriage. In the literature^9^, there is a description of a mutant homozygous case and its heterozygous sister, generated from consanguineous marriage, which is different from the present study. And, in agreement with the present data, the mutation, in hetero- or homozygosis, was also not found in all 108 control patients in the literature^9^. Regarding the 1246C-T mutation, in the present study, the wild homozygous CC genotype was more prevalent in the Control Group and the mutant TT genotype was found only in Cases Group I (with Anemia). The comparison between Cases Group I (with Anemia) and Cases Group II (without Anemia) revealed that the wild C allele was significantly associated with a reduction factor to the chance of anemia and the mutant allele T was significantly associated with the chance of presenting anemia. In the literature^8^, this mutation was found in compound heterozygosis (1246C-T/3_5delCTT) in an investigated patient, of non-consanguineous marriage, and in heterozygosis, in his mother, only for the 1246C-T mutation. The study^8^ showed that in the paternal parent and in all 50 controls, the mutation was not found, unlike the present study in which it was found, in heterozygosis, in the control group, which was composed of a sample twice as large.

The discovery of other genes involved in iron deficiency, such as STEAP3/TSAP6^7^ or TMPRSS6^12^, or new mutations in already identified genes, such as the N491S and G212V mutations, both in the *SLC11A2* ^1^ gene, suggests that these conditions may be more frequent than initially assumed and the clinical diagnosis of these conditions should be based on the first set of hematological and serum iron evaluation, followed by genetic testing, as performed in the present study. The molecular approach to IRIDA after bariatric surgery, excluding other possible etiologies, as performed in this present study, may provide new perspectives and direction for implementing more personalized recommendations for iron supplementation. Genetic expression patterns can be a predictive tool for responsiveness to nutritional treatments, and the knowledge of these mutations may have relevance on the clinical and therapeutic approach, in order to personalize their management and follow-up.

Despite the positive results found in the present study, they must be interpreted with caution and need to be corroborated by multicentric and/or independent studies to determine the real prevalence of both studied mutations in the *SLC11A2* gene and its association with IRIDA after RYGB in the general population and also in the Brazilian population. Furthermore, genetic anemia has similarities with anemia of chronic diseases or that after bariatric surgery, and a differential diagnosis between these diagnostic hypotheses requires careful investigation. The diagnosis of IRIDA also requires a genetic test that is not yet available in most laboratory centers; therefore, its clinical features are not fully understood and not even diagnosed.

## CONCLUSIONS

The results suggest the association of the 1246C-T mutation, in the *SLC11A2* gene, and the etiopathogenesis of IRIDA, not responsive to supplementation, in this sample evaluated.

There are differences, at the molecular level, in patients with and without IRIDA after bariatric surgery using RYGB.
